# Biochar is an innovative strategy for reconstructing microbial communities and enhancing nutrient utilization efficiency in acidic red soils

**DOI:** 10.3389/fmicb.2025.1622408

**Published:** 2025-06-02

**Authors:** Hanfeng Jiang, Jiangyan Wu, Jialu Guan, Fangzhou Zhao, Linghua Tan, Haoming Chen

**Affiliations:** ^1^School of Environmental and Biological Engineering, Nanjing University of Science and Technology, Nanjing, China; ^2^School of Chemical Engineering, Nanjing University of Science and Technology, Nanjing, China

**Keywords:** soil improvement, carbon sequestration and emission reduction, acid regulation, plant growth, nutrient cycling

## 1 Introduction

### 1.1 Acidification of red soils: a complex global issue requiring urgent solutions

Red soils, a highly weathered soil type distributed globally across ~64 million square kilometers, primarily concentrated in tropical and subtropical regions including southern China, Southeast Asia, South America, and Africa, covering about 45.2% of the global land area (Yu et al., 2024). Globally, soil acidification has led to reduced productivity on about 50% of arable land, with local crop losses as high as 43.8% (Du et al., 2024). Low nutrient content and poor water-holding capacity are prominent characteristics of red soils under conditions of high acidification. The causes of red soil acidification are complex, driven both by natural processes and closely related to human activities. Natural acidification processes are primarily driven by the leaching of base cations (Ca^2+^, Mg^2+^, K^+^) caused by long-term high-intensity rainfall and the continuous weathering of aluminosilicate minerals (Li et al., 2025). Anthropogenic acidification, on the other hand, is primarily promoted by excessive application of chemical fertilizers (especially long-term input of nitrogen fertilizers), acid deposition, and unreasonable farming management practices, which collectively contribute to the accumulation of hydrogen ions and the activation of reactive aluminum (Lang et al., 2025).

### 1.2 Soil acidification significantly impairs the ecological functions of rhizosphere microbiota

More seriously, soil acidification can significantly alter the structure of rhizosphere microbial communities. In particular, functionally important microbes critical for plant growth (e.g., nitrogen-fixing bacteria, phosphate-solubilizing bacteria, and carbon-fixing bacteria) exhibit marked reductions in abundance and metabolic function in acidic red soils. This functional decline not only reduces the efficiency of soil nutrient cycling but also intensifies greenhouse gas emissions (Xu et al., 2025). Moreover, acidification disrupts the ecological balance of rhizosphere microbial communities, impairing the colonization ability and functional performance of beneficial microbes in the rhizosphere. Consequently, plants face difficulties in efficiently acquiring nutrients via microbial symbiotic networks and in resisting pathogen attacks. For instance, the antagonistic effect of rhizosphere microbial communities in acidic red soils against the plant pathogen *Fusarium* spp. has decreased by 20.6 to 50.7% (Li et al., 2023). Therefore, mitigating red soil acidification is essential for maintaining soil ecosystem health, promoting sustainable agriculture, and addressing climate change.

## 2 Biochar as a potential optimal green and low-carbon technology for the remediation of acidic red soils

Currently, the primary measures to address red soil acidification involve the application of organic or inorganic soil amendments, such as lime, industrial by-products, and organic wastes (Dai et al., 2017; Mao et al., 2025). These materials improve soil conditions by neutralizing acidity, increasing exchangeable calcium content, and reducing aluminum toxicity. Biochar, a carbon-rich functional material produced through the pyrolysis of biomass, boasts a wide range of feedstocks, including agricultural and forestry residues (e.g., rice husks, wood chips, straw) and organic solid wastes (e.g., livestock manure, stabilized sludge; Tomczyk et al., 2020). Compared to lime and some organic solid wastes, biochar stands out due to its alkaline properties, porous structure, chemical stability, and low environmental risk, making it an ideal candidate for modulating red soil acidification and enhancing ecological functions (Chen et al., 2023). Furthermore, biochar's stable carbon structure facilitates long-term sequestration of organic carbon, enhancing soil carbon sink capacity and mitigating greenhouse gas emissions such as CO_2_ and N_2_O (Yu et al., 2024; Fei et al., 2025). Consequently, biochar not only synergistically addresses the dual demands of red soil acidification remediation and agricultural carbon sequestration, but also provides a low-carbon technological pathway for establishing sustainable agricultural systems in red soil regions.

## 3 Biochar effectively improves the physicochemical properties of acidic red soils by mitigating acidification

Biochar, enriched with alkaline components such as alkali metals, carbonates, surface oxygen-containing functional groups, and dissolved organic matter, can directly neutralize active acids and exchangeable aluminum ions in red soils, elevating the pH to a range suitable for crop growth (Liu et al., 2025). Studies have shown that under ambient conditions of 25°C, the application of biochar derived from spent mushroom substrate can increase the pH of acidic red soils by 43% (Sarfraz et al., 2023). Moreover, biochar's hierarchical pore structure and high specific surface area enhance the cation exchange capacity (CEC) and soil organic matter (SOM) content, displacing acidic cations at soil adsorption sites and reducing the risk of leaching of base cations (Ke et al., 2022). More crucially, the oxygen-containing functional groups and organic anions present in biochar effectively anchor iron and aluminum oxides and hydroxides through protonation and deprotonation reactions. This significantly enhances the soil acid buffering capacity of acidic red soils, thereby reducing the risk of secondary acidification induced by nitrogen fertilizer application or rainfall leaching (He et al., 2022). Therefore, biochar not only addresses the superficial issue of red soil acidification but also reshapes the ecological foundation of these soils by improving their physical structure and optimizing nutrient cycling through synergistic effects.

## 4 Biochar reconstructs microbial communities and promotes the activity of functional microbes in acidic red soils

**Figure 1:**
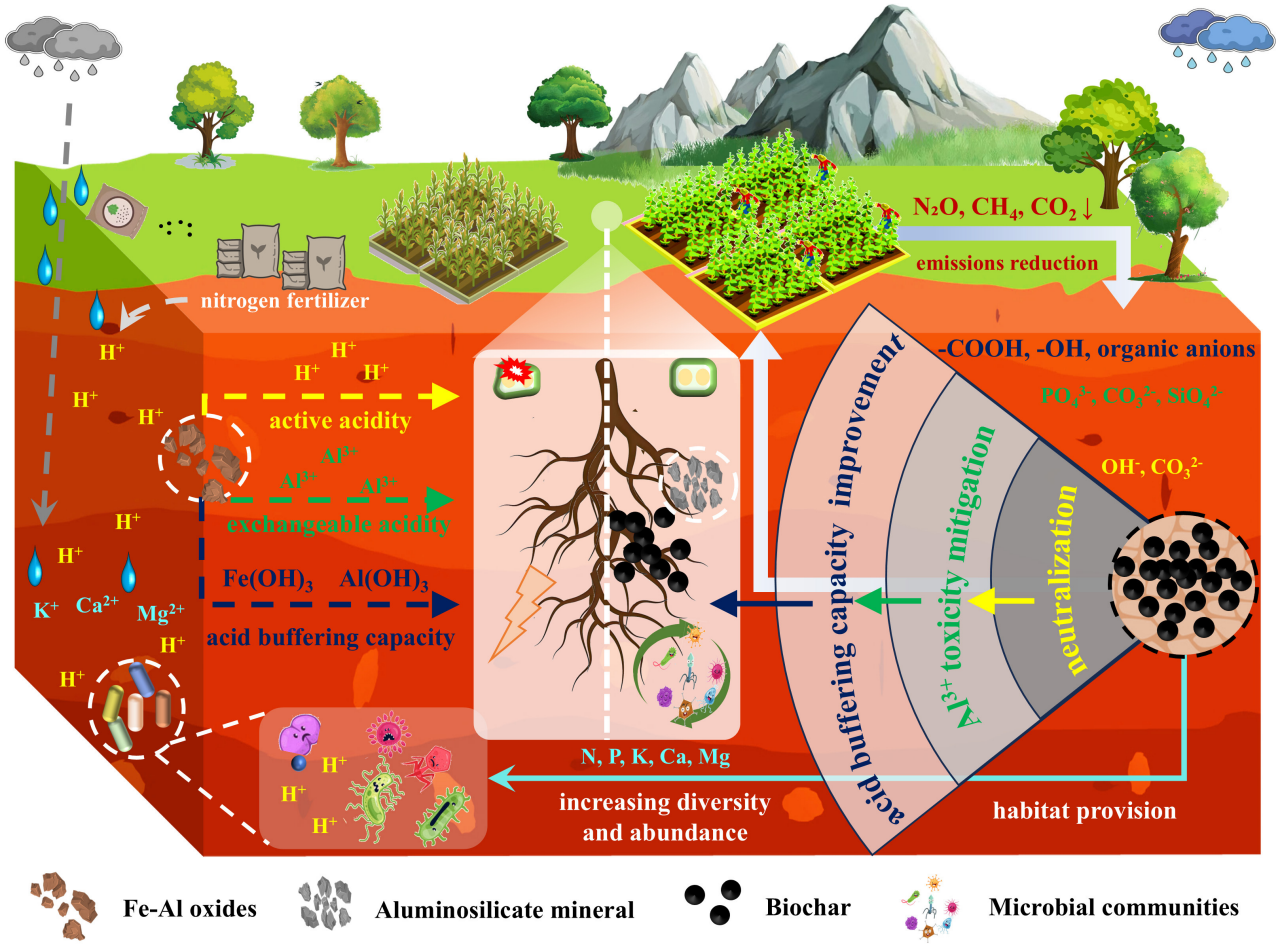
Schematic diagram of the mechanism by which biochar improves red soil acidity (neutralization, aluminum toxicity mitigation, acid buffering capacity improvement) and regulates rhizosphere microbial communities (habitat provision, increasing diversity and abundance).

Acidification and impoverishment of red soils lead to the loss of microbial diversity and functional inhibition, subsequently triggering the decline of soil ecological functions. Biochar can reconstruct microbial community structures and activate functional microbial groups through multiple mechanisms (Figure 1). Firstly, the porous structure and high specific surface area of biochar provide a protective microenvironment for microorganisms to evade acid stress. Meanwhile, the organic carbon and mineral nutrients (such as soluble phosphorus and potassium) carried on the surface of biochar can serve as electron donors and acceptors for microorganisms, promoting the colonization and proliferation of oligotrophic microorganisms (Yu et al., 2024). For instance, pig manure-derived biochar can significantly enhance microbial diversity (with a 30.08% increase in the Shannon index) and richness (with a 3.69% increase in the Chao1 index) in red soils (Yue et al., 2023). Furthermore, by elevating soil pH and reducing the concentration of reactive aluminum, biochar alleviates the metabolic inhibition of acid-sensitive bacteria such as nitrifying bacteria, while simultaneously enhancing the activity of acid-tolerant functional microbes, thereby optimizing nitrogen and phosphorus cycling efficiency (Dai et al., 2017). Notably, biochar promotes the synergistic symbiosis between functional microbial groups, such as arbuscular mycorrhizal fungi, and plants by enhancing the complexity of microbial interaction networks (e.g., symbiotic relationships and signal exchange), significantly improving the stress resistance and nutrient use efficiency of red soil systems (Yue et al., 2023). These mechanisms suggest that biochar not only reverses the negative impacts of red soil degradation on microbial communities but also activates the core service functions of red soil ecosystems through microenvironment remediation and functional regulation.

## 5 Biochar synergistically drives carbon sequestration and greenhouse gas mitigation in red soils

The addition of biochar to red soils enables carbon sequestration and reduces greenhouse gas emissions. Previous studies have demonstrated that rice straw-derived biochar can decrease the CO_2_ emission rate and cumulative emissions from red soils by 28.0 and 27.5%, respectively (Lai et al., 2024). The carbon sequestration mechanisms of biochar primarily involve direct fixation through the carbonization of indigenous organic matter and indirect fixation through interactions within the soil ecosystem. The increase in soil pH induced by biochar application suppresses microbial respiration, thereby reducing CO_2_ emissions from red soils (Yu et al., 2024). Additionally, biochar inhibits the activity of key enzymes in the nitrogen cycle (such as urease and nitrate reductase) through physical adsorption and chemical passivation, mitigating N_2_O production fluxes during nitrification and denitrification processes (Dai et al., 2017). Meanwhile, redox-active components on the surface of biochar, including quinone groups, graphitized carbon, and metal oxides, can mediate electron transfer, stimulating the metabolic pathways of anaerobic bacteria (such as methanogens and denitrifying bacteria) and reducing the emission intensities of CH_4_ and N_2_O (Fei et al., 2025). In summary, the synergistic mechanisms of carbon sequestration and emission reduction associated with biochar application can effectively alleviate carbon emissions from red soil agriculture, contributing to the sustainable management of red soils and the achievement of carbon neutrality goals.

## 6 Discussion and outlook

Despite the numerous benefits of biochar application in red soils, its efficacy remains influenced by various factors, including initial soil properties, biomass feedstocks, pyrolysis processes, and application methods. For instance, variations in biochar's pH and porosity may lead to inconsistent outcomes across different red soil regions. Therefore, it is essential to tailor feedstock selection, process optimization, functional modifications, or combined applications according to the unique characteristics of red soils in different regions.

The long-term stability of biochar in mitigating red soil acidification is significantly affected by regional climate and agricultural management practices. Intense rainfall may accelerate the leaching of alkaline substances, while continuous fertilization could alter the surface charge properties of biochar, leading to a gradual decline in amelioration effects. Additionally, biochar derived from manure may contain heavy metals (e.g., Cu, Zn) that could accumulate in soils after long-term application. Notably, while the persistent carbon structure of biochar is advantageous for carbon sequestration, its long-term interference effects on indigenous soil microbial communities are not yet fully understood. The large-scale application of biochar still necessitates a cautious evaluation of potential ecological risks and long-term impacts. Consequently, future research should focus on the targeted design of functionalized biochars, their combined applications with other technologies, and the assessment of ecological effects over extended periods. Such endeavors will facilitate the sustainable improvement and restoration of red soils.
